# Noninvasive Fecal Cytokine and Microbiota Profiles Predict Commencement of Necrotizing Enterocolitis in a Proof-of-Concept Study

**DOI:** 10.1016/j.gastha.2023.03.003

**Published:** 2023-03-07

**Authors:** Christian Zenner, Lisa Chalklen, Helena Adjei, Matthew J. Dalby, Suparna Mitra, Emma Cornwell, Alexander G. Shaw, Kathleen Sim, J. Simon Kroll, Lindsay J. Hall

**Affiliations:** 1 Intestinal Microbiome, School of Life Sciences, ZIEL – Institute for Food & Health, Technical University of Munich, Freising, Germany; 2 Gut Microbes & Health, Quadram Institute Biosciences, Norwich, United Kingdom; 3 Leeds Institute of Medical Research, University of Leeds, Leeds, United Kingdom; 4 Department of Medicine, Section of Paediatric Infectious Disease, Imperial College London, London, United Kingdom; 5 Norwich Medical School, University of East Anglia, Norwich, United Kingdom

**Keywords:** Preterm Infants, Noninvasive Biomarkers, Cytokines., Necrotizing Enterocolitis, Gut Microbiota

## Abstract

**Background and Aims:**

Necrotizing enterocolitis (NEC) is a life-threatening disease and the most common gastrointestinal emergency in premature infants. Accurate early diagnosis is challenging. Modified Bell’s staging is routinely used to guide diagnosis, but early diagnostic signs are nonspecific, potentially leading to unobserved disease progression, which is problematic given the often rapid deterioration observed. We investigated fecal cytokine levels, coupled with gut microbiota profiles, as a noninvasive method to discover specific NEC-associated signatures that can be applied as potential diagnostic markers.

**Methods:**

Premature babies born below 32 weeks of gestation were admitted to the 2-site neonatal intensive care unit (NICU) of Imperial College hospitals (St. Mary’s or Queen Charlotte’s & Chelsea) between January 2011 and December 2012. During the NICU stay, expert neonatologists grouped individuals by modified Bell’s staging (healthy, NEC1, NEC2/3) and fecal samples from diapers were collected consecutively. Microbiota profiles were assessed by 16S rRNA gene amplicon sequencing and cytokine concentrations were measured by V-Plex multiplex assays.

**Results:**

Early evaluation of microbiota profiles revealed only minor differences. However, at later time points, significant changes in microbiota composition were observed for Bacillota (adj. *P* = .0396), with *Enterococcus* being the least abundant in Bell stage 2/3 NEC. Evaluation of fecal cytokine levels revealed significantly higher concentrations of IL-1α (*P* = .045), IL-5 (*P* = .0074), and IL-10 (*P* = .032) in Bell stage 1 NEC compared to healthy individuals.

**Conclusion:**

Differences in certain fecal cytokine profiles in patients with NEC indicate their potential use as diagnostic biomarkers to facilitate earlier diagnosis. Additionally, associations between microbial and cytokine profiles contribute to improving knowledge about NEC pathogenesis.

## Introduction

Necrotizing enterocolitis (NEC) is a life-threatening disease that primarily affects very low birthweight (VLBW) preterm infants born weighing less than 1500g.^1^ The estimated average incidence of NEC cases across 27 studies conducted worldwide is ∼7% among VLBW infants.^2^ However, contrasting regional differences are reported in the literature, with a prevalence of NEC of 25.4% for enteral fed and low birthweight infants admitted to public hospitals in Addis Ababa, Ethiopia,^3^ compared to only 1.6% in VLBW infants in Japan.^4^

Although clinical manifestations of the disease have been known since the 1940s,^5^ its etiology remains incompletely understood and is often described as multifactorial.^6^ The most important contributing factor for the development of NEC is prematurity, including low birthweight and low gestational age.^7^^,^^8^ Other potential factors are formula feeding,^9^ prolonged parenteral feeding,^10^ and an abnormal microbial gut colonization,^11^ potentially leading to a perturbed state in the premature intestine.^12^^,^^13^ The gut of vaginally delivered and breast-fed term babies is typically dominated by bacteria of the genus *Bifidobacterium,*^14^^,^^15^ whereas preterm infants, who are often born by caesarean section and receive antibiotic treatment, are populated by genera such as *Enterococcus, Klebsiella,* and *Enterobacter.*^15^ Overgrowth of these potentially pathogenic bacteria within the gut microbiota, and/or colonization of the preterm gut by hospital-acquired pathogens plays a crucial role in the onset of NEC.^16^ Frequently detected bacteria occurring in association with NEC include *Clostridium* spp*., Enterococcus* spp.*, Escherichia coli, Pseudomonas aeruginosa, Salmonella* spp*., Klebsiella* spp.*, and Staphylococcus* spp. ^16^ These potential pathogens can be partially suppressed by supplementation with probiotics including *Bifidobacterium* spp. and *Lactobacillus* spp., which is also associated with a 50% reduction in NEC incidence.^17^^,^^18^

The prognosis for infants diagnosed with NEC is poor, with survivors at risk of long-term neurodevelopmental limitations and growth restrictions.^19^, ^20^, ^21^ The Bell staging criteria were introduced in 1978 to classify different stages of illness severity, suggest disease management, and guide treatment,^22^ and were later refined in 1986.^23^ Various other staging criteria for NEC have been proposed by expert neonatologists, including the Vermont Oxford Network definition, Centers for Disease Control and Prevention definition, Gestational Age-Specific Case Definition of NEC, 2 of 3 rule, Stanford NEC score, and International Neonatal Consortium NEC workgroup definition. However, modified Bell staging remains the most frequently used,^24^ despite questions remaining about its reliability.^25^

Researchers have focused on additional measures including the infant gut microbiota that could better predict cases of NEC. Dobbler et al reported that both lower microbial diversity and bacteria belonging to the family *Enterobacteriaceae* correlated with NEC, with *Citrobacter koseri* and *Klebsiella pneumoniae* being the most abundant species within this family.^26^ Low bacterial diversity in combination with high abundance of Pseudomonadota prior to the onset or at diagnosis of NEC has been confirmed by other studies.^27^, ^28^, ^29^, ^30^, ^31^, ^32^, ^33^ In contrast, Cassir et al showed a strong association between *Clostridium butyricum* and NEC incidence and identified cytotoxic activity in the supernatant of cultured C. *butyricum* isolates.^34^ The role of the gut microbiota in the development of NEC remains complex and is likely to be dependent on NICU location (i.e. circulating nosocomial pathogens) and underlying individual microbial communities present in the preterm infant gut.

Human milk oligosaccharides (HMOs) are now a topic of research interest due to their role in feeding specific bacteria, especially *Bifidobacterium*, which are not typically abundant in the preterm infant gut microbiota.^35^ Sodhi et al recently suggested the HMOs 2′-fucosyllactose and 6′-sialylactose protect against the development of NEC through the inhibition of Toll-like receptor (TLR) 4 signaling.^36^ Masi et al showed that the concentration of the HMO disialyllacto-N-tetraose was lower in the breast milk of mothers of NEC infants and associated with a lower abundance of *Bifidobacterium* species.^37^

The role of cytokines and pro-inflammatory mediators in NEC has been extensively reviewed. In particular, increased levels of TLR 4, interleukin (IL)-18, interferon (IFN)-γ, platelet-activating factor, IL-6, IL-8, IL-1β, and nuclear factor-κB have been linked to NEC severity, while deficiencies of TLR 9, IL-1R8, IL-1Ra, transforming growth factor (TGF)-β_2_, platelet-activating factor-acetylhydrolase, and IL-10 pave the way for NEC-associated inflammation.^38^

Novel approaches are needed to provide guidance to clinicians and healthcare professionals to select the appropriate therapy.^39^ Previous studies have aimed to find suitable and robust biomarkers that may be used to predict NEC, including platelet counts,^40^ levels of C-reactive protein,^41^ serum amyloid A,^42^ claudin proteins,^43^ plasma citrulline,^44^^,^^45^ endogenous RNA molecules,^46^ volatile organic compounds,^47^ lipocalin-2, and calprotectin.^48^ Systemic cytokine concentrations have been suggested as potential biomarkers for the prediction of NEC and disease outcome.^38^^,^^49^, ^50^, ^51^, ^52^ Rising cytokine levels were highly specific for the diagnosis of neonatal sepsis, but additional (noninvasively assayed) biomarkers are needed for high specificity and sensitivity to predict NEC.^53^

In this study, we evaluate the gut microbiota profiles and the measurement of fecal cytokine levels as a rapid and noninvasive tool for the early detection of NEC.

## Methods

### Study design

Samples were provided from a study published in 2015.^13^ This exploratory study included infants born <32 weeks of gestation, without severe congenital birth defects. Infants were admitted to the Imperial College Healthcare National Health Service Trust neonatal intensive care unit (NICU) between January 2011 and December 2012. In total, 39 individuals were included in the study (Bell stage 1: n = 7; Bell stage 2/3: n = 11; healthy controls: n = 21). Probiotics and H2-receptor antagonists were not used within the NICU at the time of recruitment and sampling. Patient identification numbers were blinded. Only members of this research group had access to patient information.

### Sample collection

Research nurses collected fecal samples from diapers using a sterile spatula, placed in sterile DNase-, RNase-free Eppendorf tubes, stored in a −20 °C freezer on the neonatal unit within 2 hours of collection, and stored at −80 °C within 5 days. NEC cases were diagnosed by the attending neonatal consultant and confirmed by an independent neonatologist (Bell stage 2/3 by Bells’ modified staging criteria). Multiple samples were taken from individuals included in the study during their stay in NICU. Sample numbers were as follows: Bell stage 1 NEC n = 23; Bell stage 2/3 NEC n = 47; healthy controls n = 86.

### Cytokine measurement

One gram of fecal material was homogenized with one ml phosphate-buffered-saline (PBS) using a FastPrep bead beater (4.0m/s, 3min), centrifuged (14,000rpm, 15min) and 25μl of supernatant was used for the assay. Samples were analyzed using MULTI-SPOT plates, MESO Quickplex SQ120 and discovery workbench software according to the manufacturer’s protocol. Pre-coated immunoassays V-PLEX Proinflammatory Panel 1 (human) and V-PLEX Cytokine Panel 1 (human) were used to detect a set of 20 different cytokines: IFNγ, IL-1β, IL-2, IL-4, IL-6, IL-8, IL-10, IL-12p70, IL-13, tumor necrosis factor (TNF)α, granulocyte-macrophage colony-stimulating factor (GMCSF), IL-1α, IL-5, IL-7, IL-12p40, IL-15, IL-16, IL-17A, TNFβ, and vascular endothelial growth factor (VEGF)-A. If cytokine values drastically exceeded comparable sample values, the sample was excluded from the analysis. Samples not reaching the lower limit of detection were generally considered as very low and were taken into account without statistical resolving.

### DNA extraction, 16S rRNA gene amplification, and sequencing

Information about sample preparation, gene amplification, and sequencing is documented elsewhere.^13^

### 16S rRNA sequencing data analysis

Roche 454 pyrosequencing data in standard flowgram format were transcribed to fastq format using Bio.SeqIO.SffIO module in biopython. Single fastq files were remultiplexed using the perl script remultiplexor (available at https://www.imngs.org). Remultiplexed sequencing data were processed with the integrated microbial NGS platform,^54^ with parameters set as follows: Barcode mismatches, 1; quality trim score, 10; min. read length 100bp; max. read length 1000bp; max. rate of expected error, 2% of sequence length; min. alignment id 70%. Operational taxonomic units (OTUs) were clustered at 97% sequence similarity, using a cutoff of ≥0.25% relative abundance in at least one sample. Data were further analyzed and visualized using RHEA,^55^ a modular pipeline for microbial profiling, using R(v4.0.5) and Rstudio (v1.4.1106). Samples not achieving specific quality control criteria (>1000 reads/sample; rarefaction curves Figure A1) were excluded from the analysis, leading to reduced sample numbers: Bell 1 NEC 1 n = 18; Bell 2 NEC 2/3 n = 41; healthy controls n = 63.

### Statistical testing

Cytokine profiles were evaluated pairwise between groups using Mann-Whitney U test. The following methods were applied for 16S rRNA gene amplicon data: Fisher’s exact test, Wilcoxon rank sum, and Kruskal-Wallis rank sum test. The method used is referenced in the respective paragraph or figure. Multidimensional scaling plots are based on generalized UniFrac distances. The *P*-values were calculated using permutational multivariate analysis of variance.

All authors had access to the study data and had reviewed and approved the final manuscript.

## Results

A total of 39 preterm infants with a gestational age <32 weeks were included in this study, 7 were diagnosed with Bell stage 1 NEC, 11 were diagnosed with Bell stage 2/3 NEC, and 21 were healthy controls (not diagnosed with NEC). Detailed information about participants and sample numbers is represented in Table 1. All but 2 babies received a first course of antibiotics from birth onward. Fecal samples from diapers were collected longitudinally during their NICU stay.

**Table:** Cohort Information of Study Participants

Study characteristics	All	NEC2/3	NEC1	Healthy
Number of individuals	39	11	7	21
Received antibiotics	37	11	7	19
Received additional formula feeding	6	1	3	2
Received mechanical ventilation	25	10	4	11
DOL at NEC diagnosis (mean, min-max)	-	29 (9–43)	29 (17–82)	-
Samples used for microbiota analysis >1000 reads	122	41	18	63
Samples used for cytokine analysis	156	47	23	86
Gestational age (mean ± StDev)	27 + 1 (190d) ± 2 + 1 (15d)	26 + 6 (188d) ± 2 + 1 (15d)	27 + 2 (191d) ± 0 + 5 (5d)	27 + 2 (191d) ± 2 + 4 (18d)
Birthweight (mean ± StDev)	922g ± 283g	843g ± 204g	937g ± 140g	959g ± 348g
Gender	f = 15 m = 24	f = 4 m = 7	f = 2 m = 5	f = 9 m = 12

**Figure 1:**
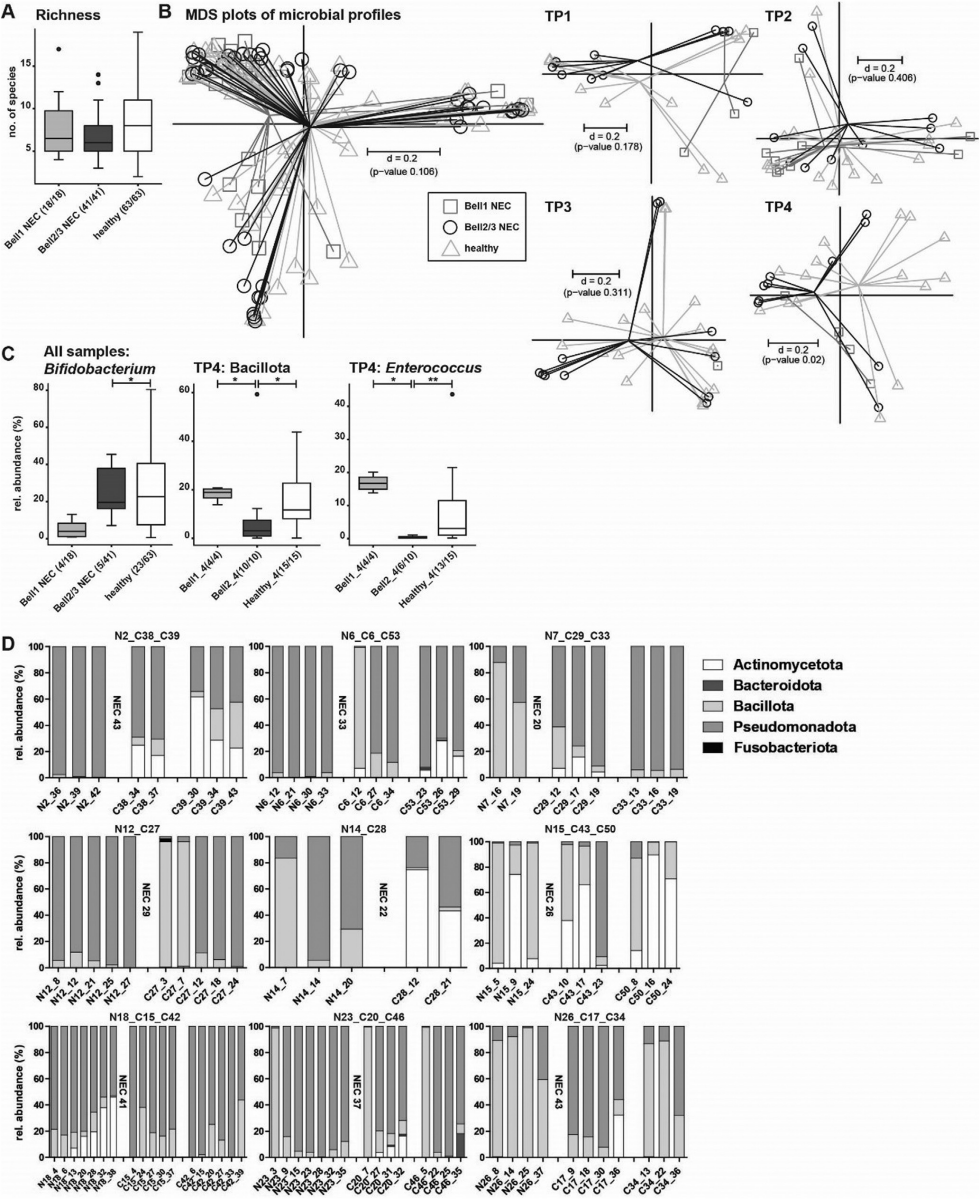
A: Alpha-diversity shown as richness. B: Inter-sample differences shown as multidimensional scaling plots based on generalized UniFrac distances across all samples and separated by different time points. C: Taxonomic differences across all samples (*Bifidobacterium*) and at time point 4. Numbers in brackets indicate the number of samples positive for the observation. D: Over time age-matched taxonomic profiles at the phylum level of preterm babies that developed NEC (left) compared to healthy individuals (right). The DOL of NEC diagnosis is indicated after NEC samples. *P*-value summary: ∗<0.05; ∗∗<0.01.

Characterization of the neonatal gut microbiome of these preterm infants was carried out using 16S rRNA gene amplicon sequencing. An average of 7.8 (±3.6) OTUs (a proxy for bacterial species) was detected across the 3 infant groups. Healthy infants contained a mean of 8.4 OTUs/sample, which was lower at 7.6 OTUs/sample in the NEC1 infants and 6.9 OTUs/sample in the NEC2/3 infants, but the differences were not statistically significant (Figure 1A). The multidimensional scaling plot of microbial profiles representing *beta*-diversity showed no significant differences across the 3 study groups (*P* = .106) (Figure 1B). To detect age-dependent differences, samples were split up into 4 different time points (TP1: 0–10 days of life (DOL), TP2: 11-20 DOL, TP3: 21-30 DOL, TP4: 31-maximum age). Significant differences in the *beta*-diversity were detected at time point 4 in the multidimensional scaling plot (*P* = .02) (Figure 1B). By comparing the groups at taxonomic levels, the only detected significant differences were between Bell stage 2/3 and healthy controls for the order Bifidobacteriales, including family Bifidobacteriaceae and genus *Bifidobacterium* (adj. *P* = .0204 for all 3 taxonomic levels, Fisher’s exact test, pairwise comparison) (Figure 1C).

At all taxonomic levels, no significant differences were detected at TP1 and TP2. At TP3, a significantly higher relative abundance of *Escherichia-Shigella* in Bell 2/3 was detected compared to the healthy group (*P* = .0003, Wilcoxon rank sum test, pairwise, data not shown). At TP4, the microbiota profiles became more clearly different. The phylum Bacillota was lower in Bell 2/3 (mean rel. abundance 10.0 %) compared to Bell 1 (mean rel. abundance 18.1%) and healthy (mean rel. abundance 15.6%) (adj. *P* = .0396, Wilcoxon rank sum test, pairwise comparison, equal *P*-value for both comparisons) (Figure 1C). Differences in Bacillota were mostly represented by differences in the family Enterococcaceae and the subordinate genus *Enterococcus* (NEC1 vs NEC2/3 adj. *P* = .0142; NEC2/3 vs healthy adj. *P* = .0096, Wilcoxon rank sum test, pairwise, values are equal for family and genus) (Figure 1C). Individuals that developed NEC were further compared with age-matched healthy preterm babies, with phylum profiles measured longitudinally until NEC diagnosis. Only 2 NEC babies displayed high Actinomycetota abundance (N15, N18), whilst this phylum was better represented in the healthy control babies. Bacteroidota was generally underrepresented in the studied individuals. Fusobacteria were also rare and only found in one control baby at one time point (C27_3) (Figure 1D).

We also explored factors that could potentially impact microbiota profiles, for example, condition at birth (Appearance, Pulse, Grimace, Activity, Respiration,), total parenteral nutrition (TPN), need for mechanical ventilation, feeding type, and antibiotics usage. Appearance, Pulse, Grimace, Activity, Respiration score can be used as prognostic indicator for neonatal death in preterm infants,^56^ however, differences between study groups were minor and not significant. TPN was performed for all but 4 babies, and has been previously shown to impact the gut microbiota.^57^ In this study, all samples were taken after TPN (average length of TPN NEC1 5.3 days, NEC2/3 5.7 days, healthy 6.1 days) was finished, thus we were not able to determine if there were any TPN-associated microbial changes. The need for mechanical ventilation was heterogeneous across all groups. We did not observe any significant differences in NEC1 and NEC2/3 groups. However, within the healthy group, and only analyzing samples between 9 and 21 DOL to reduce the age bias, microbial richness was significantly elevated in the non-ventilated group (*P* = .0087). In terms of feeding type, only 6 individuals (NEC2/3 n = 1; NEC1 n = 3; healthy n = 2) received formula milk (‘top-up’) in addition to maternal and/or donor breast milk, and we did not observed any clear differences. We did observe some changes in one individual in the Bell stage 1 group, from 12 DOL to 14 DOL during formula feeding (rise of Actinomycetota by 5%, an increase of Pseudomonadota by 13%, and a decrease of Bacillota by 18%), however, this is only one individual and these changes may be associated with normal microbiota changes over time. Regarding antibiotics usage, only 2 individuals (both in the healthy group) did not receive antibiotics during their NICU stay, which correlated with high abundance of Actinomycetota (genus *Bifidobacterium,* at TP2 and TP3).

Fecal cytokine concentrations were then analyzed to determine differences in these host-associated immune factors. Pro- and anti-inflammatory cytokines play an important role in the development and progression of NEC and systemic levels are often measured. As NEC is essentially an intestinal disease, cytokine concentrations measured in feces could be more representative of immune activation in NEC.

**Figure 2:**
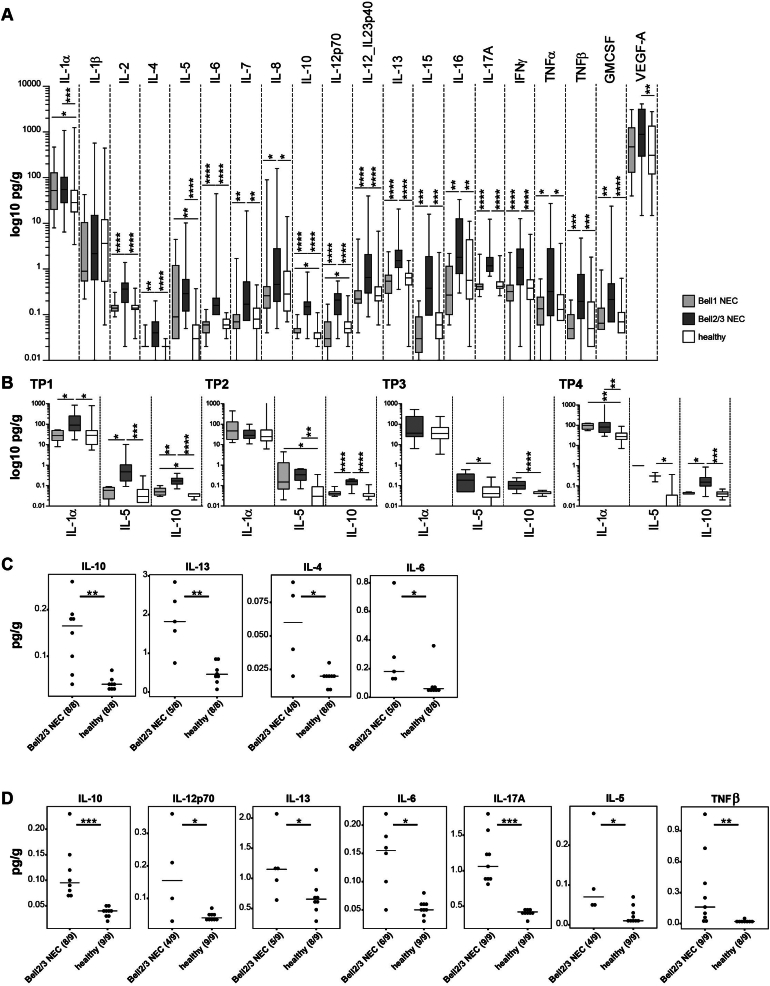
Cytokine levels measured in fecal samples of preterm infants in the 3 study groups: Bell stage 1 NEC, Bell stage 2/3 NEC, and healthy. A: Across all time points. B: Divided by time points for IL-1α, IL-5, and IL-10. At TP3, as only one sample was present in the NEC 1 group, it was excluded from the analysis. Concentrations in pg/g are plotted on a log 10 scale for better visibility. C: Significant cytokines 5–10 days before NEC diagnosis compared to age-matched controls. D: Significant cytokines 11–17 days before NEC diagnosis compared to age-matched controls. Numbers in brackets indicate the number of samples (one per individual) positive for the observation (if NA was reported, the number of samples is reduced). Comparisons for panels A and B were statistically analyzed with Mann-Whitney U test. Comparisons for panels C and D were statistically analyzed with Wilcoxon rank sum test. *P*-value summary: ∗<0.05; ∗∗<0.01; ∗∗∗<0.001; ∗∗∗*∗<0.0001.*

In these infants almost all measured cytokine concentrations were significantly higher in the NEC 2/3 group (Figure 2A). Significant differences between NEC1 and NEC2/3 as well as between NEC2/3 and healthy were observed for IL-2, IL-6, IL-10, IL-12p70, IL-12_IL23p40, IL-13, IL-17A, and IFNγ (*P* ≤ .0001, Mann-Whitney U test) (Figure 2A). Significantly higher concentrations in NEC1 compared to healthy were observed for IL-1α (*P* = .045), IL-10 (*P* = .032), and IL-5 (*P* = .0074), suggesting that these could be potential markers for the onset and development of NEC. The concentration of these cytokines was further investigated at each time point (Figure 2B). For IL-1α, significant differences were detected at TP1 (NEC1 vs NEC2/3, *P* = .0307, and healthy vs NEC2/3, *P* = .0177) and TP4 (healthy vs NEC1, *P* = .0057, and healthy vs NEC2/3, *P* = .001). For IL-5, significant differences were observed at TP1 (NEC1 vs NEC2/3, *P* = .0106, and healthy vs NEC2/3, *P* = .0004), TP2 (healthy vs NEC1, *P* = .0115, and healthy vs NEC2/3, *P* = .004), TP3 (healthy vs NEC2/3, *P* = .0228), and TP4 (healthy vs NEC2/3, *P* = .0432). Significantly higher levels of IL-10 were found in the NEC2/3 group at all time points, TP1 (NEC1 vs NEC2/3, *P* = .0045, healthy vs NEC1, *P* = .031, healthy vs NEC2/3, *P* < .0001), TP2 (NEC1 vs NEC2/3, *P* < .0001, healthy vs NEC2/3, *P* < .0001), TP3 (healthy vs NEC2/3, *P* < .0001), and TP4 (NEC1 vs NEC2/3, *P* = .0275, healthy vs NEC2/3, *P* = .0003).

Cytokine profiles were further analyzed 5–10 days before the date that NEC was diagnosed and compared with age-matched healthy preterm infants ( ± 1 day difference). Significantly higher levels of IL-10 (*P* = .0013), IL-13 (*P* = .0062), IL-4 (*P* = .0293), and IL-6 (*P* = .0322) were measured in the Bell stage 2/3 group compared to healthy controls (Figure 2C). The same analysis was performed 11–17 days before NEC diagnosis with significant differences again detected for IL-10 (*P* = .0004), IL-13 (*P* = .0335), and IL-6 (*P* = .0122), with additional cytokines IL-12p70 (*P* = .0294), IL-17A (*P* = .0004), IL-5 (*P* = .0294), and TNFβ (*P* = .0066) also differentiating between NEC and healthy controls (Figure 2D).

When we analyzed cytokine profiles with additional clinical variables (as outlined above), we only observed significant differences for mechanical ventilation within the healthy group (samples between 9 and 21 DOL were analyzed to reduce the age bias) for IL-15, which was significantly higher in the ventilated group (*P* = .0427).

## Discussion

Although known for decades, NEC remains a major challenge for neonatologists, given the abrupt onset and rapid progression of the disease. Targeted treatments are still lacking, leading to high mortality rates and leaving survivors with severe long-term disabilities. Prompt timing of treatment is crucial to maximize the chance of survival. In this study, we investigated the preterm infant gut microbiota in combination with fecal cytokine levels to shed light on disease progression. The preterm intestinal microbiota differs greatly from that of term infants: the number of species present is reduced, patterns of colonization are disrupted, and the abundance of pathogenic bacteria is increased.^58^, ^59^, ^60^ Many studies have reported that reduced gut bacterial diversity is a risk factor for the onset of NEC.^26^^,^^28^^,^^31^^,^^61^ In our study, samples from the NEC 2/3 group contained the lowest number of OTUs per sample (mean of 6.9), but compared to the other study groups differences were minor and not significant. In terms of taxonomic differences, an enrichment of Pseudomonadota and a reduction of Bacillota and Bacteroidota have often been associated with NEC development.^12^^,^^62^ However, this was not observed in our study results, with similarly high levels of Pseudomonadota found in all study groups. We did detect significantly lower levels of Bacillota in NEC 2/3 infants at time point (TP4), representing higher Pseudomonadota levels, but this was only the case for infants older than 31 days and was not associated with NEC.

A variety of reasons could account for differences between studies, including sampling technique, DNA extraction protocols, selection of 16S variable regions, sequencing technique, bioinformatics pipelines, and databases used,^63^^,^^64^ making comparisons between studies difficult. As numerous bacteria are potentially associated with NEC, that is, *Clostridium* spp., *Enterococcus* spp*., Escherichia coli, Pseudomonas aeruginosa, Salmonella* spp*., Klebsiella* spp.*, and Staphylococcus* spp.,^16^ a single bacterial signature is not expected. On the other hand, supplementation of probiotic *Bifidobacterium* and *Lactobacillus* is associated with lower abundance of common pathobionts in the preterm gut,^17^ which is associated with significantly reduced rates of NEC and late onset sepsis.^18^ Indeed, we also observed healthy preterms had higher relative abundance of *Bifidobacterium*, when compared to NEC 2/3 infants, even though these infants did not receive probiotic supplementation. Exploring additional clinical factors revealed that only mechanical ventilation significantly impacted microbial diversity, but this was only observed in ‘healthy’ premature infants. Surprisingly, we did not see any major differences in formula feeding or antibiotic usage, which would be expected to significantly alter microbiota profiles. This is most likely linked to the low number of formula fed babies, and the fact all were still receiving breast milk thus masking any potential diet-induced changes,^65^ and although we observed higher *Bifidobacterium* (which is highly susceptible to antibiotics) in nonantibiotic treated preterms, this was only 2 infants. Given the limited number of patients and samples, this restricted our ability to do multiple robust comparisons across key clinical parameters.

Although substantial differences in microbiota profiles were not found in this study between NEC infants and healthy controls, the impact of the microbiota on the immune system, including signaling molecules such as cytokines, is well-known.^66^ Therefore, the evaluation of fecal cytokine levels is a key aspect of this study. Interestingly, except for IL-1β, the fecal concentrations of all measured cytokines were significantly higher in the NEC 2/3 group compared to healthy controls. IL-1s (including IL-1α and IL-1β) are pro-inflammatory cytokines, produced by a variety of cell types that also induce inflammatory reactions such as tissue damage and fever.^67^ IL-1 receptor binding triggers the activation of pro-inflammatory transcription factors such as NF-κB and AP-1, which can further induce the production of IL-6, TNF, and IL-1 itself.^67^ Studies on human IL-1α and IL-1β in NEC setting are rare. One study by Benkoe et al could not identify differences in systemic IL-1β levels in NEC babies compared to healthy controls,^49^ concordant with the results of our study. For IL-1α, we could identify significantly higher levels in NEC2/3 compared to NEC1 and healthy at TP1, and significantly higher levels in NEC2/3 and NEC1 compared to healthy at TP1 (Figure 2B). Interestingly, this finding did not persist during TP2 and TP3, and was again observed at TP4. However, this may be due to the inconsistent number of samples across all time points, which is a limitation of this proof-of-concept study. Another study by Ng et al showed increased systemic concentrations of IL-2, IL-4, IL-6, IL-10, IFNγ, and TNFα in neonatal septicemia, also including NEC cases,^68^ corresponding with the results presented in this study for fecal cytokines. We could also show that local IL-10 levels were significantly higher in NEC2/3 compared to NEC1 and healthy at all-time points (Figure 2B). Additionally, the age-matched comparison of babies 5–10 or 11–17 days before NEC diagnosis revealed significantly higher levels of IL-10 (Figure 2D), indicating an induced protective role of IL-10 to counteract inflammation in the gut. This is also supported by high levels of IL-10 in breast milk,^69^ while low levels of IL-10 in breast milk are correlated with NEC incidence.^70^ IL-5 primarily promotes activation, survival and adhesion of eosinophils, and is therefore elevated in allergy and parasitosis.^71^ Interestingly, we observed significantly higher IL-5 concentrations in NEC2/3 at all-time points (Figure 2B), suggesting a hyper-inflammatory state with involvement of eosinophils, coinciding with a study from 2000.^72^ While IL-4 and IL-5 were involved in NEC progression in rats,^73^ Benkoe et al demonstrated significantly lower IL-4 and IL-5 concentrations in NEC serum samples compared to healthy controls.^49^ Although we explored a set of twenty different cytokines, we may have missed additional and important cytokines involved in NEC onset/development. Indeed, recently it was shown that transgenic IL-37 may prevent dysregulation of adaptive immunity in murine NEC, and that this cytokine modulates immune homeostasis.^74^

We acknowledge as this a twin-center site proof-of-concept study with a limited number of individuals (and longitudinal samples), this is a limitation. A larger multicenter study, with, for example, a greater divergence in clinical care regimens, may allow additional key differences to be teased apart, but this was not possible in our limited study. Furthermore, samples were sequenced in 2014 and could not be re-sequenced due to a lack of sufficient material, which may have impacted our microbiota data. Shotgun metagenomic sequencing could provide more specific results including at the species and functional level thus providing a more comprehensive overview of microbiota changes prior to NEC onset. Moreover, relative stool hydration could have influenced the protein content in fecal samples and thus, affected overall cytokine measurements. For this reason, standardization of input material before subjection to cytokine measurement may enhance robustness and accuracy in further studies.

## Conclusion

These findings suggest that fecal cytokine concentrations could provide additional measures in the diagnosis of NEC. Particularly IL-1α, IL-10, and IL-5, which show a rise from healthy to NEC 1 and to NEC2/3 could potentially be used as accessory markers to the current Bell staging that is routinely performed. The timing of sampling and a rapid analysis yielding results within 24 hours would be essential for the most effective use of fecal cytokine measurement in aiding the diagnosis of NEC. Our data indicate that profiling fecal cytokine levels, particularly IL-5 and IL-10, from 14 days onward, and regular testing every third day for increasing levels could act as a predictive test, warning of developing NEC, but this needs to be confirmed in a larger, multicenter study. Furthermore, robust reference values of healthy preterm infants and other NEC cases from other NICUs will be required to define highly selective and sensitive cytokine thresholds, in order to provide additional information and guidance to neonatologists in the diagnosis of NEC. Additional research will also need to test and validate different platforms for fecal cytokine analysis, and compare different preterm infant cohorts to explore cytokine profile variation across different NICUs, as robust markers would be key for next-stage studies. Although further testing is required, development of an early diagnosis could refine therapeutic measures, mitigate disease outcomes, increase survival rates, and reduce long-term consequences for survivors.

## Supplementary materials

Figure A1

Table A2
