# Supplementary figures and images for: Noninvasive Fecal Cytokine and Microbiota Profiles Predict Commencement of Necrotizing Enterocolitis in a Proof-of-Concept Study
Source: Gastro Hep Adv. 2023 Mar 7;2(5):666–75. doi: 10.1016/j.gastha.2023.03.003 (PMC10352139; doi:10.1016/j.gastha.2023.03.003)

Rarefaction curve of all samples

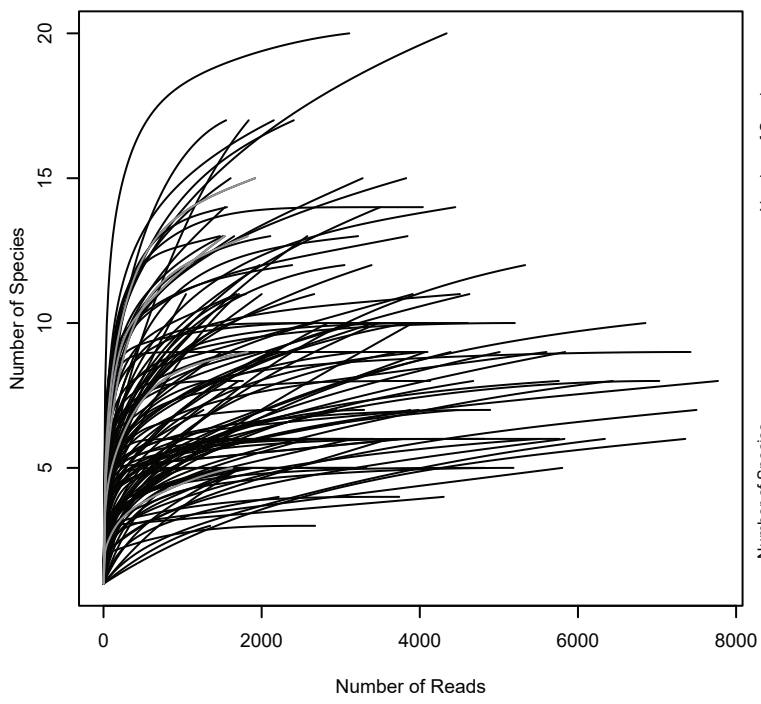

Rarefaction curves of samples at different timepoints

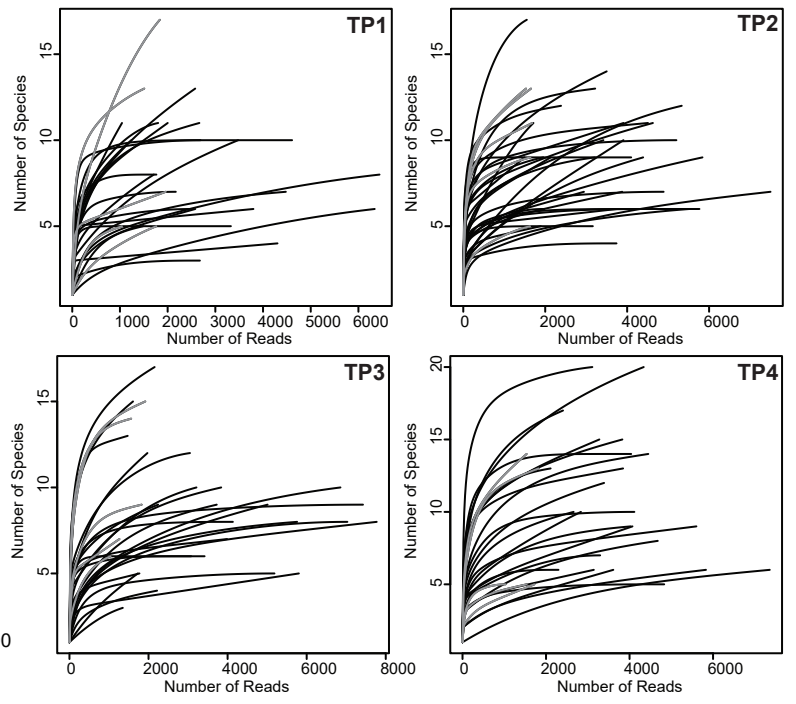

Supplement: Figure A1 [file mmc1.pdf]
